# Metagenomic analysis of viral genes integrated in whole genome sequencing data of Thai patients with Brugada syndrome

**DOI:** 10.5808/gi.22047

**Published:** 2022-12-30

**Authors:** Suwalak Chitcharoen, Chureerat Phokaew, John Mauleekoonphairoj, Apichai Khongphatthanayothin, Boosamas Sutjaporn, Pharawee Wandee, Yong Poovorawan, Koonlawee Nademanee, Sunchai Payungporn

**Affiliations:** 1 Program in Bioinformatics and Computational Biology, Graduate School, Chulalongkorn University, Bangkok 10330, Thailand; 2 Research Unit of Systems Microbiology, Department of Biochemistry, Faculty of Medicine, Chulalongkorn University, Bangkok 10330, Thailand; 3 Center of Excellence for Medical Genomics, Medical Genomics Cluster, Faculty of Medicine, Chulalongkorn University, Bangkok 10330, Thailand; 4 Excellence Center for Genomics and Precision Medicine, King Chulalongkorn Memorial Hospital, The Thai Red Cross Society, Bangkok 10330, Thailand; 5 Research Affairs, Faculty of Medicine, Chulalongkorn University, Bangkok 10330, Thailand; 6 Department of Medicine, Faculty of Medicine, Center of Excellence in Arrhythmia Research Chulalongkorn University, Chulalongkorn University, Bangkok 10330, Thailand; 7 Interdisciplinary Program of Biomedical Sciences, Graduate School, Chulalongkorn University, Bangkok 10330, Thailand; 8 Division of Cardiology, Department of Pediatrics, Faculty of Medicine, Chulalongkorn University, Bangkok 10330, Thailand; 9 Bangkok General Hospital, Bangkok 10330, Thailand; 10 Department of Pediatrics, Faculty of Medicine, Chulalongkorn University, Bangkok 10330, Thailand; 11 Department of Medicine, Faculty of Medicine, Chulalongkorn University, Bangkok 10330, Thailand; 12 Pacific Rim Electrophysiology Research Institute, Bumrungrad Hospital, Bangkok 10110, Thailand

**Keywords:** Brugada syndrome, human endogenous retrovirus K, metagenome, VIRIN, virus integration breakpoint, whole genome sequencing

## Abstract

Brugada syndrome (BS) is an autosomal dominant inheritance cardiac arrhythmia disorder associated with sudden death in young adults. Thailand has the highest prevalence of BS worldwide, and over 60% of patients with BS still have unclear disease etiology. Here, we performed a new viral metagenome analysis pipeline called VIRIN and validated it with whole genome sequencing (WGS) data of HeLa cell lines and hepatocellular carcinoma. Then the VIRIN pipeline was applied to identify viral integration positions from unmapped WGS data of Thai males, including 100 BS patients (case) and 100 controls. Even though the sample preparation had no viral enrichment step, we can identify several virus genes from our analysis pipeline. The predominance of human endogenous retrovirus K (HERV-K) viruses was found in both cases and controls by blastn and blastx analysis. This study is the first report on the full-length HERV-K assembled genomes in the Thai population. Furthermore, the HERV-K integration breakpoint positions were validated and compared between the case and control datasets. Interestingly, Brugada cases contained HERV-K integration breakpoints at promoters five times more often than controls. Overall, the highlight of this study is the BS-specific HERV-K breakpoint positions that were found at the gene coding region "*NBPF11*" (n = 9), "*NBPF12*" (n = 8) and long non-coding RNA (lncRNA) "*PCAT14*" (n = 4) region. The genes and the lncRNA have been reported to be associated with congenital heart and arterial diseases. These findings provide another aspect of the BS etiology associated with viral genome integrations within the human genome.

## Introduction

Brugada syndrome is an inherited arrhythmogenic disease leading to a high risk of acute cardiac death. This syndrome has been connected to a genetic variant with an autosomal dominant inheritance pattern [1, 2]. The highest prevalence is in Southeast Asia, especially in Thailand (6.8 per 1,000), where it is almost 15 times higher than worldwide [2-4]. Hundreds of gene variants have been associated with Brugada syndrome, of which the mutation in *SCN5A* or *SCN10A* genes of sodium channels has been commonly found with the disease (30%) [5,6]. While new findings, the *MAPRE2* mechanism, and the microtubule-related trafficking effects on NaV1.5 expression have been explored in Brugada syndrome by genome-wide association analysis [5], for almost 60% of patients, etiologic causes are still unknown [7].

The whole genome sequencing (WGS) data have become highly valuable information that can be used to screen all variants, allele assignment, insertion, and detection of structural variation [8,9]. Generally, after mapping WGS reads to the human reference genome, there remains 5%–10% unmapped reads [10]. The unmapped reads may contain microbial agents, especially viral elements, due to the integrative capacity of various viruses [11]. The metagenomics approach is suitable for identifying uncharacterized sequencing reads [12].

Approximately 8% of the human genome contains human endogenous retroviruses. The human endogenous retrovirus (HERV)’s transcripts and regulatory functions have been identified in numerous diseases [13,14] including multiple sclerosis [15], diabetes [16], systemic lupus erythematosus [17], psoriasis [18], rheumatoid arthritis [19], and cancer [20]. Moreover, human papillomavirus (HPV), hepatitis B virus (HBV), and Epstein‐Barr virus (EBV) are exogenous viruses that are associated with diseases. They are well-known as insertion viruses commonly found in the human genome and can induce tumorigenesis and cancer (10%–15% of all cancer) [21,22]. Local viral integrations may cause genomic instability followed by altered gene copy numbers and gene expression around the integration sites. Therefore, these inserted positions provide valuable information for understanding the mechanisms of virus-related diseases and the etiologic [23].

Additionally, the infections such as enteroviruses (coxsackievirus, enterovirus, echovirus) and adenoviruses play an important role in sudden cardiac death [24,25]. The most common cardiotropic viruses are EBV, coxsackievirus, adenovirus, human herpesvirus 6 (HHV6), cytomegalovirus, hepatitis C virus, and parvovirus B19. Moreover, parvovirus B19 was also associated with Brugada syndrome. They potentially trigger an autoimmune response against components of the heart or mediate direct cardiac injury [26]. Thus, viral genes and integration positions in Brugada syndrome patients are useful evidence that can be used to discover the disease's etiology and progression.

We identified the putative viral gene and protein in 200 Thai male WGS data. We further developed an analysis pipeline to identify virus integration positions in human genome sequencing data called "VIRIN". The human endogenous retrovirus K (HERV-K) genomes were assembled and explored in two potential integration loci of HERV-K, namely the "*neuroblastoma breakpoint genes family* (*NBPF*)" gene family and long non-coding RNA (lncRNA) "*PCAT14*", which are related to Brugada syndrome from WGS data of an individual Thai patient with Brugada syndrome and a control volunteer.

## Methods

### Study cohort

The study cohort was divided into two groups, the Brugada cases and controls. The cases consisted of 100 Thai male subjects with type I Brugada electrocardiogram (ECG) using the criteria of the 2013 Heart Rhythm Society/European Heart Rhythm Association/Asia Pacific Heart Rhythm Society Expert Consensus Statement. The training physicians read and confirmed all ECGs of cases with a type I Brugada pattern. The 100 Thai male control subjects comprised those who had a standard 12-lead ECG without type I Brugada. Both groups were representative of a wide age range (between 19 and 75 years, with medians of 50 years in the case group and 47 years in the control group). All subjects were of Thai ethnicity by self-report. The Ethics Review Committee of all the institutions approved the study (NCT04232787).

All blood samples were collected, and DNA was extracted as described in a previous report. The sequencing libraries were prepared with a polymerase chain reaction–free reaction [9]]. Then, the human genomic DNA libraries were sequenced by Illumina HiseqX platforms (Cambridge, UK) with a pair-end sequencing (2 × 150 bp) strategy [9]].

### Extraction of the unmapped and soft-clipped sequence

First, the raw FASTQ reads were filtered with Trimmomatic version 0.38 [27] by sliding window at mapping quality 30; all reads shorter than 50 nucleotides were removed. Second, the filtered reads were mapped to the NCBI Genome Reference Consortium Human Build 38 (GRCh38) with decoys reference using iSAAC-03.16.02.19 (version 0.7.16a) with a default setting. An unmapped read whose mate is mapped was extracted using the SAMtools 1.15 "view -f 4 -F 264" command [28]. Furthermore, the soft-clipped reads with GRCh38 were extracted using the modified extractSoftclipped function of the SE-MEI tools (https://github.com/dpryan79/SE-MEI).

### Identification of the viral sequences

The viral sequence identification was performed on the NCBI 13,434 complete reference viral database (https://ftp.ncbi.nlm.nih.gov/refseq/release/viral/, downloaded on January 7, 2021) by the Bowtie2 aligner version 2.3.5.1 [29]. Next, the identified viral sequence was merged by *de novo* assembly approach utilizing SPAdes v3.13.0 with the "-k 33" option [30]. Then, to identify the viral integration site, the contigs were validated with the blastn in NCBI BLAST+ [31] and Diamond blastx v2.0.15.153 [32], respectively.

The original extracted single-mate unmapped and soft-clipped reads were re-aligned with the selected virus reference genome. The reads mapped with the virus reference genome were extracted from the original GRCh38 human alignment bam file. The position of the virus integration was reported by the in-house bash script and BEDtools v2.27.1 command [33]. The virus integration positions (breakpoints) were annotated with DNase I hypersensitivity regions, Repeatmasker, and GencodeV.40 [34-36]. The whole sequence of steps of the analytical source code is available on GitHub (https://gist.github.com/Suwalak-Chit/VIRIN).

### Data interpterion and statistical analysis

Statistical analysis and visualization were performed using GraphPad Prism 8.0.1 software. The descriptive statistics and continuous variables consistent with a normal distribution were represented by means and standard deviations; non-parametric tests were performed with t-tests or the Mann-Whitney U test. p < 0.05 was considered statistically significant.

### Data availability

The data in this study were available from the National Research Council of Thailand under license for the current study and were not publicly available. The data were available from the authors upon a reasonable request and with the permission of the National Research Council of Thailand.

## Results

### The characterization of unmapped reads

**Fig. 1.**
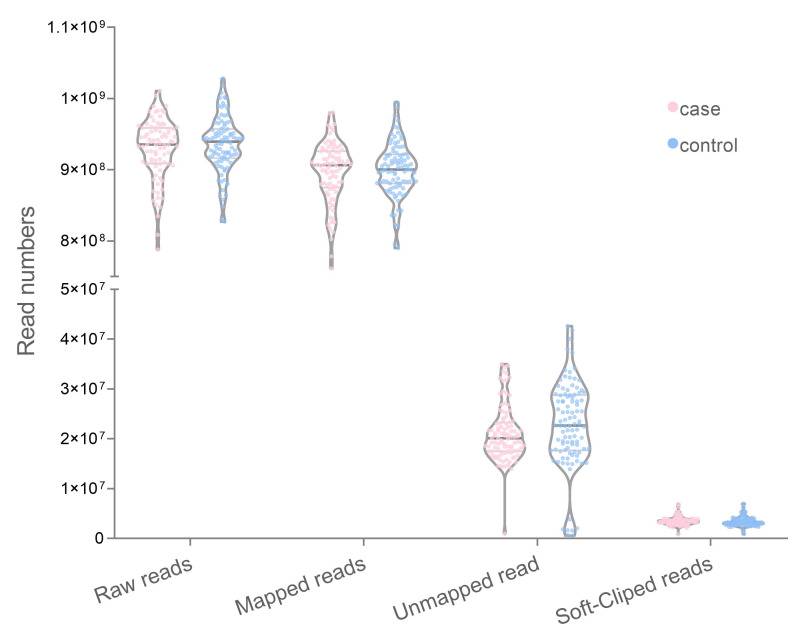
Distribution number of mapped and unmapped reads with GRCh38.

The WGS data from both case (n = 100) and control (n = 100) datasets are 929,286,470 and 935,775,065 pair-end read, respectively. Most reads were aligned with the human reference genome 96.48% (case) and 96.62% (control). The average unmapped reads which remained amounted to 44,548,377 reads (case) and 44,784,395 reads (control). Among the mapped reads, the soft-clipped reads were 9,009,042 in the case group and 8,860,802 in the control group (Fig. 1). The number of unmapped reads between groups did not significantly differ in the t-test (p = 0.172).

### Viral gene or/and genome identification

**Fig. 2.**
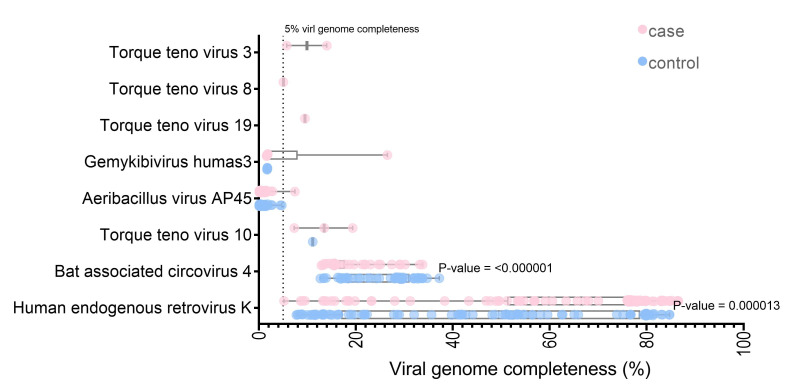
The longest contigs size of each identified virus by nucleotide blast from individual sample.

The unmapped and soft-clipped reads were aligned against the NCBI viral genome using Bowtie2. With a cut-off of at least 1,000 reads containing 90 viruses in each sample, 291,126,786 reads were assigned to the 285 viral references. The SPAdes assembly tools [37] were used for *de novo* assembly of the virus mappable read in each sample. The whole contigs were hit with eight virus genomes (the contig length >5% of each virus genome) by blastn. Three viruses (Torque teno virus 10 [TTV 10], human endogenous retrovirus K, and Bat associated circovirus 4) were found in both datasets. Five viruses (Torque teno virus 19, Torque teno virus 3, Torque teno virus 8, Aeribacillus virus AP45, and Gemykibivirus humas3) were found only in the case dataset. Seven of the eight identified are DNA viruses (Supplementary Table 1). Interestingly, the HERV-K and Bat-associated circovirus four genome completeness significantly differed between the cases and controls (p < 0.001) (Fig. 2). Almost full-length genome of human endogenous retrovirus K was observed for the datasets (the largest contig in case, 86.54%; control, 84.74% genome completeness) (Fig. 2).

**Fig. 3.**
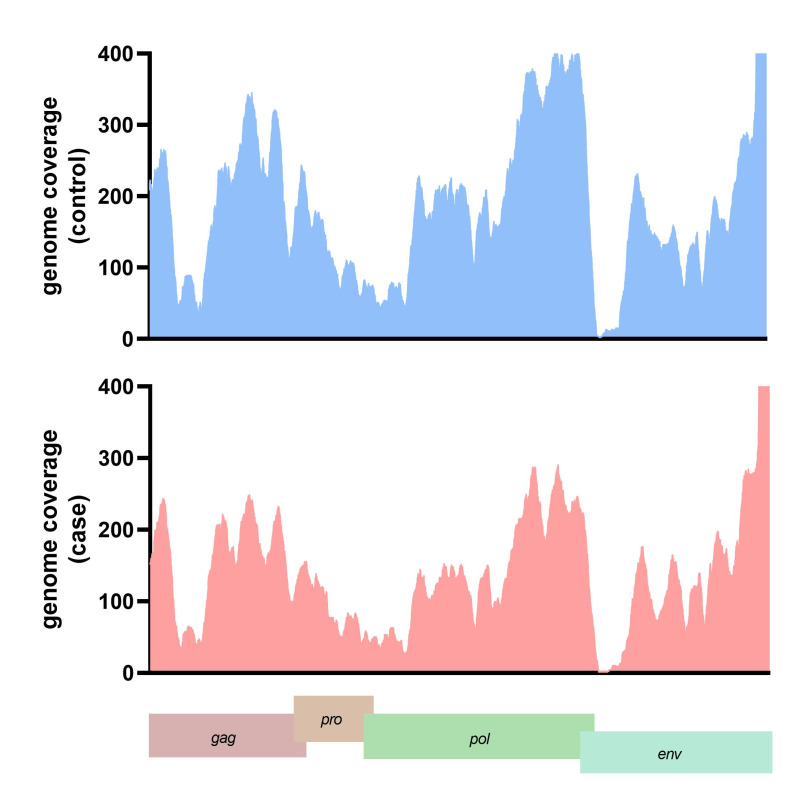
The genome depth coverage of human endogenous retrovirus K (HERV-K) in representative case and control.

The translated nucleotide blast on protein database (Blastx) result showed 13 viral proteins positive in more than ten samples in each group. The human endogenous retrovirus K putative envelope protein was detected in all subjects from the blastx results (Supplementary Table 2). The HERV-K genome was assembled and the genome coverage was visualized. For the case subject data, the genome depth coverages on each HERV-K gene were gag gene (194.44x), pro gene (410.79x), pol gene (203.53x), and env gene (189.76x), respectively. In contrast, the genome coverage of HERV-K genes in the control subject data included the gag gene (199.44x), pro gene (610.79x), pol gene (243.53x), and env gene (129.76x) (Fig. 3). We also detected the A55 protein of the BeAn virus genome in 61 cases and 70 control data. Moreover, 11 from 13 (84.61%) of the identified viral proteins were retrovirus proteins.

### VIRIN analysis pipeline validation

The viral breakpoint integration position was identified with the VIRIN analysis pipeline. The pipeline was validated with the cervical cancer cells (HeLa cells) and hepatocellular carcinoma (HCC) tissue WGS data, in which well-known HPV-18 and HBV are integrated into the cellular genome, respectively [38]. The result from VIRIN showed HPV-18 was integrated into the three breakpoints of locus 8q24.21 (Chr8:127,222,011, Chr8:127,218,387 and Chr8: 127,229,303) of HeLa cell WGS data (SRR5009881) [39]. Moreover, the analysis pipeline identified two HBV breakpoints at locus 17p13.1 (Chr17:10,110,360 and Chr17:10,366,141) from HCC tissue WGS data (ERR173408 and ERR181167) [40] (Supplementary Table 3). This evidence suggests that VIRIN is effective for the identification of virus integration breakpoints.

### HERV-K breakpoint position identification

**Fig. 4.**
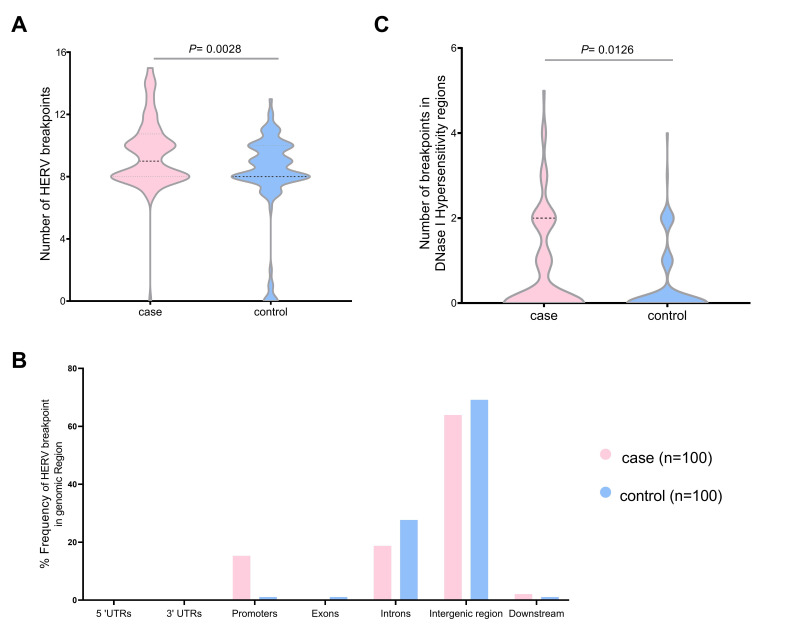
The number of human endogenous retrovirus K (HERV-K) breakpoints in the human genome individual case (n = 100) and control group (n = 100) HERV-K breakpoints (A), the total distribution of HERV-K breakpoints in the genomic region (B), and the individual HERV-K breakpoints in DNase I hyposensitivity regions (C). UTR, untranslated region.

We validated the HERV-K breakpoints in the human genome. The total breakpoints were 952 in the case group and 814 in the control group datasets. The number of breakpoints was significantly different between the two datasets (Mann-Whitney U test, p = 0.0028) (Fig. 4A). Chromosome 1 had the highest HERV-K breakpoints in both datasets (case, 38; control, 20), while chromosomes 13, 15, and 20 had no HERV-K breakpoint position. Chromosomes 2, 12, 16, and X contained the HERV-K breakpoints only in the case dataset (4, 3, 8, and 7, respectively). However, there were no significant differences (t-test, p < 0.05) in HERV-K insertion in any chromosomes between the case and control (Supplementary Table 4). Moreover, the number of HERV-K breakpoints in DNase I hypersensitivity regions were significantly different between case group (81 breakpoints) and control group (40 breakpoints) (Mann-Whitney U test, p = 0.012) (Fig. 4B). More than 60% of HERV-K breakpoints were located in the intergenic region (case, 63.89%; control, 69.15%). The breakpoint in case datasets was more often in promoter than control datasets (15.28% and 1.06%, respectively) (Fig. 4C). The HERV-K breakpoints at the repeat region include 54 and 26 breakpoints from 30 cases and 20 controls, respectively. HERV-K breakpoints in the case group (32 breakpoints) were located mostly on the long terminal repeat (LTR), while most HERV-K breakpoints from the control group (12 breakpoints) were located on the long interspersed nuclear element (LINE) (Table 1).

All HERV-K breakpoints were annotated on the gene (gencodeV.40), including 27 positions from 27 cases and 29 positions from 18 controls. The HERV-K breakpoints were located in protein-coding (case, 17; control, 20), lncRNA (case, 8; control, 7) and a small number of pseudogene (case, 2; control, 3) (Supplementary Table 5). An individual unique HERV-K breakpoint was also found in 16 cases and 21 controls. Importantly, 25 HERV-K breakpoints from nine Brugada syndrome cases were located in a *NBPF*. Additionally, the HERV-K breakpoint from four Brugada syndrome samples was located on the lncRNA name *PCAT14* (prostate cancer-associated transcript 14). Even though the HERV-K breakpoint in the nicotinamide nucleotide transhydrogenase function (*NNT*) gene was found in many samples, more were detected in the control dataset (n = 10) than in the case dataset (n = 5). Meanwhile the *NBPF11*, *NBPF12*, and *PCAT14* were found only in the case group, and not detected in the control dataset (Tables 2 and 3).

## Discussion

### Viral gene/genome detection

A viral metagenome analysis was performed on the Brugada syndrome cases and control WGS data, to investigate the viral genome integration from the WGS unmapped read in each sample. Our data analysis found the genes of 90 integrated viruses from both human and non-human viruses. The human viruses in this study were members of the *Herpesviridae* family and HERV, the same as in previous reports [12,41]. Almost 80% of virus-aligned reads were assigned as dsDNA virus because the library preparation kit was appropriated for DNA source, which similar to the result of virome in human WGS from the previous study [42]. According to the library preparation approach, giant viruses (large genome viruses) such as the Pandoravirus (2 Mbp genome size) were observed in our study as well as in the previous report [43].

The *de novo* assembled contigs were aligned with the virus reference genomes (NCBI). Eight viruses were kept after excluding the virus contigs that contained less than 5% of their genome. TTV, a dsDNA virus belonging to the *Anelloviridae* family, had considerable genetic variability and extreme diversity [44]. In previous reports, the TTV DNA was detected in secretions of healthy humans, such as blood, saliva, breast milk, tears, bile, and urine [45,46]. The TTVs DNA level was also considered the marker of the immunological status, hepatitis, gastroenteritis, periodontitis, multiple sclerosis, and cancer [47,48]. In this study, we have found a unique TTV19, TTV8 in a specific case, TTV3 in two cases, and TTV10 in both groups (case, 3; control, 1). Nevertheless, several virome studies of human blood frequently found TTV in the samples [49], while we reported only eight samples in total. Indeed, we found TTV reads in a total of 20 samples; however, some TTV contigs' number, coverage and length are too low. Since the human WGS library preparation has a virus enrichment step, we found TTV in a few samples. However, according to our findings, we could not confirm that TTV integration could be related to Brugada syndrome. On the other hand, the non-human viruses were known as the contaminant DNA from the reagents or sequencing process, including bacteriophage (Aeribacillus virus AP45) and mammalian virus (Bat associated circovirus four and Gemykibivirus humas3) [50]. The previously frequently found viruses associated with a cardiac infection, such as HHV6, EBV, and hepatitis C virus were also detected but could not be assembled to the large contigs [26]. Even though Parvoviruses B19 was reported as being associated with Brugada syndrome, it was not found in our data.

### VIRIN validation

Previous research has reported that three virus segments of HPV18 are integrated into the HeLa genome on chromosome 8 (locus 8q23-24) upstream of the *myc* gene [51]. The result of VIRIN analysis pipelines was found in three HPV-18 integrated positions in the locus 8q23.21 of HeLa cell line WGS data. Generally, the HBV integration breakpoints in HCC are various. Most HBV breakpoints are near coding genes, including the *TERT*, *MLL4*, *CCNE1*, *SENP5*, and *ROCK1*. Recurrent HBV breakpoints occur within or close to repetitive, non-coding sequences, such as LINEs, Alu short interspersed elements, and LTR [52]. Our result also showed HBV integrated into the coding region of the *MYH13* gene at 17p13.1 in HCC tissue WGS data, similar to a previous report [53]. Thus, the VIRIN analysis pipeline can identify virus integration in human WGS data.

### HERV-K breakpoint positions

In addition, we can assemble the full-length HERV-K genome in all datasets. The translated nucleotide blast on protein databases (blastx) result showed that the envelope proteins (env) of HERV-K were found in all datasets. In our data analysis, we also detected known DNA proviruses, including gag, pro, pol, and env, of HERV-K [54]. Since we utilized the WGS data for the blast, our data were not a direct viral protein identification. This is the first study demonstrating the HERV-K breakpoints in the WGS data of the Thai population. The polymorphic integration position of HERV-K could influence both viral protein production and host gene regulation. The specific HERV-K breakpoints might be associated with the potential pathogenicity in different individuals, for example, neurologic and immunologic diseases [54].

DNase I hypersensitivity regions are the important genomic landmarks for functionally active open chromatin. A previous report also showed that 15% of HERVs inserts are in the DNase I hypersensitivity region [55-57]. Interestingly, our results showed higher HERV-K breakpoints at DNase I hypersensitivity regions in the case group compared to the control group. Moreover, the breakpoints of HERV-K were located at the promoter regions 15 times more often in the case group compared to the control group, and this might be linked to the gene regulation process of Brugada syndrome pathogenesis. Moreover, Brugada syndrome is 8-10 times more prevalent in men than women [58], and our result showed HERV-K was integrated at chrX only in the case group (7 cases and 0 control). The previous studies also found a variant on the *KCNJ5* (potassium voltage-gated channel subfamily E regulatory subunit 5) gene located on chromosome X in Japanese patients with Brugada syndrome [59,60]. However, our result did not find HERV-K breakpoints in any gene regions. The potential of sex hormones for cardiac regulation is through the ion channel because the cardiac muscle found the main gonadal steroid receptors. Moreover, many ion channels, such as *CACNA1C* and *SCN5A*, are very sensitive to testosterone, and this could explain the gender difference in the prevalence of Brugada syndrome [61,62]. The highest number of HERV-K breakpoints is in the LTR from all the repeated regions in both groups. Although the HERV-K were generally integrated into the LTR regions, the number of the HERV-K breakpoints in the case group LTR region was five times higher than in the control group [63].

Importantly, our data found that the HERV-K breakpoints were in the protein-coding and lncRNA region. HERV-K breakpoints within the *NNT* gene were found in many samples (control, 10; case, 5). NNT is a proton pump in the inner mitochondrial membrane found throughout the human body. It is highly expressed in the adrenals, bladder, kidneys, thyroid, adipose tissue, and especially in the heart [64,65]. Several reports showed that the lack of the *NNT* gene triggers the down-regulation of glucocorticoid levels, inhibiting cardiovascular conditions. This finding is linked to our study that found more HERV-K breakpoints in the *NNT* gene in the control group than in the Brugada syndrome patient group [66,67]. However, the gene expression level of NNT protein in the heart plays an important role in the pathogenesis; thus, NNT protein levels in Brugada syndrome are interesting and need further investigation.

Most of the HERV-K breakpoints in the gene region were the *NBPF* genes, which were implicated in several developmental and neurogenetic diseases and congenital heart disease [17,68]. Furthermore, the NBPF family, such as NBPF1 and NBPF11, were reported as the translocation disrupts a sodium channels gene on chromosome 17 called *ACCN1* (amiloride-sensitive cation channel 1) [69,70]. Even if the mechanism of the *NBPF* gene family to electrophysiology is still unknown, it is possible that the HERV-K breakpoints on *NBPF* genes are related to Brugada syndrome pathogenesis. However, the underlying mechanism needs to be further investigated.

Several lncRNAs play an important role in cardiovascular diseases, such as lncRNA *LIPCAR* "dysregulation in heart failure and lncRNA *MIAT* upregulations in myocardial infarction [71-73]. A lncRNA gene, *PCAT14*, plays an important role in tumorigenesis in HCC and prostate cancer [74,75]. *PCAT14* expression is also an important prognostic for predicting metastatic disease. Furthermore, *PCAT14* contains the single nucleotide variants of SNP rs73155085-A and rs131408-C. The rs73155085-A and rs131408-C have been reported to be associated with coronary artery disease and peripheral arterial disease, respectively [17,76]. Thus, the breakpoint at the *PCAT14* gene is potentially involved with the Brugada syndrome pathogenesis. Moreover, *PCAT14* has been associated with the hormone testosterone in prostate cancer [77].

In conclusion, some key findings have emerged from this work. The HERV-K genome and their breakpoints in the Thai population genome have been reported, and the HERV-K breakpoint positions have been found in the data. Two (*NBPF* gene and *PCAT14* lncRNA) of these breakpoints have a reasonable potential to be key pathogenesis features of Brugada syndrome. Hence, these findings provide a new viewpoint on the etiology of Brugada syndrome, including the association with viruses and virus integration positions, and not limited to purely human genetics.

## Figures and Tables

**Table 1.** The number of breakpoints in four types of repeat regions from each dataset

Repeat region	Case (n = 32)	Control (n = 20)
LTR	32	6
LINE	15	12
Simple repeat	3	0
Retroposon	3	7
SINE	1	1
Total	54	26

LTR, long terminal repeat; LINE, long interspersed nuclear element; SINE, short interspersed nuclear element.

**Table 2.** Gene annotation list in breakpoints of HERV-K integration in case dataset

No.	Gene name	Gene type	Chr	Position	Frequency
1	*NBPF12*	Protein coding	1	146948791	8
				146949149	
				146949314	
2	*NBPF11*	Protein coding	1	148137980	9
3	*SIL1*	Protein coding	5	139112931	1
4	*NNT*	Protein coding	5	43665587	5
				43665608	
				43665610	
				43665611	
5	*PDE10A*	Protein coding	6	165500565	1
6	*SSBP1*	Protein coding	7	141752231	1
7	*TCP11L1*	Protein coding	11	33049984	1
8	*ASRGL1*	Protein coding	11	62378218	2
				62378902	
9	*PPTC7*	Protein coding	12	110571019	1
10	*ZNF140*	Protein coding	12	133093110	1
11	*MAPK1*	Protein coding	22	21848137	1
12	*TMEM51-AS1*	lncRNA	1	15135632	1
13	*NEPRO-AS1*	lncRNA	3	113025862	1
14	*LINC02614*	lncRNA	3	125896808	1
15	*ENSG00000272462*	lncRNA	6	25999489	1
16	*ENSG00000285784*	lncRNA	9	11895136	1
17	*PCAT14*	lncRNA	22	23541467	4
				23541739	
				23541740	
18	*PDCL3P4*	Pseudogene	3	101695806	4
19	*ENPP7P15*	Pseudogene	11	3451290	1

HERV-K, human endogenous retrovirus K.

**Table 3.** Gene annotation list in breakpoints of HERV-K integration in control dataset

No.	Gene name	Gene type	Chr	Position	Frequency
1	*PRDX1*	Protein coding	1	45514062	1
2	*IPP*	Protein coding	1	45739955	1
3	*MPZL1*	Protein coding	1	167771343	1
4	*CR1*	Protein coding	1	207636105	1
5	*CR1*	Protein coding	1	207637031	1
6	*NNT*	Protein coding	5	43665299	10
				43665592	
				43665597	
				43665598	
				43665601	
				43665607	
				43665608	
				43665612	
				43665614	
7	*IQGAP2*	Protein coding	5	76550644	1
8	*ARMT1*	Protein coding	6	151456042	1
9	*LHFPL3*	Protein coding	7	104750438	1
10	*ABCC2*	Protein coding	10	99827666	1
11	*TTC5*	Protein coding	14	20269289	1
12	*SLC47A1*	Protein coding	17	19505358	1
13	*ENSG00000285988*	lncRNA	10	6830418	1
14	*ENSG00000255947*	lncRNA	11	61655408	1
15	*ENSG00000259048*	lncRNA	14	38118365	1
16	*ENSG00000287879*	lncRNA	18	79960352	1
17	*ENSG00000283907*	lncRNA	19	35575243	1
18	*ENSG00000286667*	lncRNA	19	386213	1
19	*MIR548XHG*	lncRNA	21	18568413	1
20	*SEC22B4P*	Pseudogene	1	146381378	1
21	*PDCL3P4*	Pseudogene	3	101693108	1

HERV-K, human endogenous retrovirus K; lncRNA, long non-coding RNA.

## Supplementary Materials

Supplementary Table S1. The number of sample and taxonomy of viruses detected by blastn

Supplementary Table S2. The number of samples, viral protein, and species of viruses detected by blastx

Supplementary Table S3. Analysis of virus breakpoints based on WGS data of HeLa and HCC using VIRIN pipeline

Supplementary Table S4. The number of breakpoints in each chromosome for case and control dataset

Supplementary Table S5. The number of breakpoints in three types of gene coding from each dataset
