# Supplementary material for: Metagenomic analysis of viral genes integrated in whole genome sequencing data of Thai patients with Brugada syndrome
Source: Genomics Inform. 2022 Dec 30;20(4):e44. doi: 10.5808/gi.22047 (PMC9847385; doi:10.5808/gi.22047)
Supplement: Supplementary Table S1. — The number of sample and taxonomy of viruses detected by blastn [file gi-22047suppl1.pdf]

Supplementary Table 1. The number of sample and taxonomy of viruses detected by blastn

Accession No.	Species	Genus	Family	Genome	Case	Control
NC_014078.1	Torque teno virus 19	Alphatorquevirus	*Anelloviridae*	ssDNA(–)	1	-
NC_014081.1	Torque teno virus 3	Alphatorquevirus	*Anelloviridae*	ssDNA(–)	2	-
NC_014084.1	Torque teno virus 8	Alphatorquevirus	*Anelloviridae*	ssDNA(–)	1	-
NC_026809.1	Gemykibivirus humas3	Gemykibivirus	*Genomoviridae*	ssDNA	1	-
NC_048651.1	Aeribacillus virus AP45	Kamchatkavirus	*Siphoviridae*	dsDNA	1	-
NC_014076.1	Torque teno virus 10	Alphatorquevirus	*Anelloviridae*	ssDNA(–)	3	1
NC_022518.1	Human endogenous retrovirus K	-	*Retroviridae*	ssRNA-RT	100	100
NC_028045.1	Bat associated circovirus 4	Circovirus	*Circoviridae*	ssDNA(+/–)	59	45

The contig of each virus from individually sample had the genome completeness more than 5% of each viral genome.
