# Supplementary material for: Metagenomic analysis of viral genes integrated in whole genome sequencing data of Thai patients with Brugada syndrome
Source: Genomics Inform. 2022 Dec 30;20(4):e44. doi: 10.5808/gi.22047 (PMC9847385; doi:10.5808/gi.22047)
Supplement: Supplementary Table S2. — The number of samples, viral protein, and species of viruses detected by blastx [file gi-22047suppl2.pdf]

Supplementary Table 2. The number of samples, viral protein, and species of viruses detected by blastx

Accession	Species	Protein	Case	Control
YP_008603282.1	Human endogenous retrovirus K	Putative env	100	100
YP_009329619.1	BeAn 58058 virus	Kelch repeat and BTB domain-containing protein A55	61	70
YP_009243641.1	Bovine retrovirus CH15	Putative viral DNA polymerase	24	57
NP_955564.1	Mouse mammary tumor virus	p30DU-p13PR-RT-IN	10	25
NP_076572.1	Bovine gammaherpesvirus 4	Viral beta-1,6-N-acetylglucosaminyltransferase	8	23
YP_001165471.3	Woolly monkey sarcoma virus	Hypothetical protein	10	22
YP_009509095.1	Hardy-Zuckerman feline sarcoma virus	v-kit protein (370 aa) (1242 is 3rd base in codon)	11	22
NP_955595.1	Abelson murine leukemia virus	ABL	18	20
NP_041259.1	Squirrel monkey retrovirus	Gag protein	11	18
YP_009513211.1	Koala retrovirus	Pol protein	5	15
NP_041185.1	Jaagsiekte sheep retrovirus	Neutral protease large subunit	4	14
YP_628286.1	Y73 sarcoma virus	Protein-tyrosine kinase	9	12
YP_241114.1	Ovine enzootic nasal tumor virus	Gap-pro-pol fusion	-	10

The table show only viral proteins that found more than 10 samples in each dataset.
