# Supplementary material for: Metagenomic analysis of viral genes integrated in whole genome sequencing data of Thai patients with Brugada syndrome
Source: Genomics Inform. 2022 Dec 30;20(4):e44. doi: 10.5808/gi.22047 (PMC9847385; doi:10.5808/gi.22047)
Supplement: Supplementary Table S3. — Analysis of virus breakpoints based on WGS data of HeLa and HCC using VIRIN pipeline [file gi-22047suppl3.pdf]

Supplementary Table 3. Analysis of virus breakpoints based on WGS data of HeLa and HCC using VIRIN pipeline

Sample	Accession No.	No. of reads	Chr	Position	Virus	Species	Family
Hela	SRR5009881	82	8	127,222,011<br>127,218,387<br>127,229,303	NC_001357.1	Alphapapillomavirus 7 (or Human papillomavirus 18)	*Papillomaviridae*
HCC	ERR173408 and ERR181167	27	17	10,110,360<br>10,366,141	NC_003977.2	Hepatitis B virus	*Hepadnaviridae*

WGS, whole genome sequencing; HCC, hepatocellular carcinoma.
