# Supplementary material for: Metagenomic analysis of viral genes integrated in whole genome sequencing data of Thai patients with Brugada syndrome
Source: Genomics Inform. 2022 Dec 30;20(4):e44. doi: 10.5808/gi.22047 (PMC9847385; doi:10.5808/gi.22047)
Supplement: Supplementary Table S4. — The number of breakpoints in each chromosome for case and control dataset [file gi-22047suppl4.pdf]

Supplementary Table 4. The number of breakpoints in each chromosome for case and control dataset

Chromosome	No. of cases	No. of controls	p-value	Adjusted p-value
chr1	38	20	0.030215	0.604300
chr2	4	0	0.009284	0.185683
chr3	6	4	0.518825	>0.999999
chr4	10	6	0.299526	>0.999999
chr5	12	23	0.153207	>0.999999
chr6	5	1	0.154132	>0.999999
chr7	1	1	>0.999999	>0.999999
chr8	15	11	0.402872	>0.999999
chr9	1	1	>0.999999	>0.999999
chr10	1	5	0.098256	>0.999999
chr11	4	1	0.175944	>0.999999
chr12	3	0	0.081699	>0.999999
chr13	0	0	NA	NA
chr14	2	2	>0.999999	>0.999999
chr15	0	0	NA	NA
chr16	8	0	0.003741	0.074812
chr17	1	1	>0.999999	>0.999999
chr18	0	1	0.318531	>0.999999
chr19	3	4	0.761852	>0.999999
chr20	0	0	NA	NA
chr21	0	1	0.318531	>0.999999
chrX	7	0	0.006909	0.138184
chrY	0	1	0.318531	>0.999999

NA, not available.
