# Supplementary material for: Metagenomic analysis of viral genes integrated in whole genome sequencing data of Thai patients with Brugada syndrome
Source: Genomics Inform. 2022 Dec 30;20(4):e44. doi: 10.5808/gi.22047 (PMC9847385; doi:10.5808/gi.22047)
Supplement: Supplementary Table S5. — The number of breakpoints in three types of gene coding from each dataset [file gi-22047suppl5.pdf]

Supplementary Table 5. The number of breakpoints in three types of gene coding from each dataset

Gene annotation	Case (n = 27)	Control (n = 18)
Protein coding	17	20
LncRNA	8	7
Pseudogene	2	2
Total	27	29

lncRNA, long non-coding RNA.
